# ACADEMIC NURSING RESEARCH, INNOVATION, AND ENTREPRENEURSHIP

**DOI:** 10.1093/geroni/igae098.0489

**Published:** 2024-12-31

**Authors:** Marcia Shade

**Affiliations:** University of Nebraska Medical Center, Omaha, Nebraska, United States

## Abstract

Marcia Shade PhD, BS, RN is the Founder and CEO of Voice-It Inc. The start-up company focuses on creating digital health solutions that engage, inform, and empower older adults. Voice-It Inc. is harnessing, AI, the power of machine learning and natural language processing to help improve the health and quality of life of older adults. Dr. Shade will share: 1) Her perceptions on the importance of understanding entrepreneurship as an academic nurse researcher and innovator, and 2) Reflections on her experience as a finalist in the 2023 NIA Start-Up Challenge. “I received invaluable guidance from the NIA Team, experts, mentors, advisors, and fellow cohort finalists to help me grow as an entrepreneur and lay a foundation for my start-up company.”

